# Uddanam nephropathy in India: a challenge for epidemiologists

**DOI:** 10.2471/BLT.17.196758

**Published:** 2017-10-03

**Authors:** Praveen Gadde, Suresh Sanikommu, Ramesh Manumanthu, Anitha Akkaloori

**Affiliations:** a Department of Public Health Dentistry, Vishnu Dental College, Bhimavaram, West Godavari District, Andhra Pradesh, India.; b Department of Public Health Dentistry, Mallareddy Dental College for Women, Hyderabad, Telangana, India.

Uddanam is a lush green region with rich coconut and cashew plantations in Srikakulam district, Andhra Pradesh state, India. An unknown number of people living in this area have a chronic kidney disease of unknown etiology, a disease that mostly affects farmers and agricultural workers.^1^ This condition was discussed and named Uddanam nephropathy at the 2013 International Congress of Nephrology held in Hong Kong, China.^2^

An increased prevalence of chronic kidney disease has been observed in several geographical areas across the world over the past two decades.^1^ These endemic nephropathies include: mesoamerican nephropathy in the central American countries; Balkan endemic nephropathy in the Balkan states; aristolochic acid nephropathy in Belgium, China and other countries that use herbal medicines; Sri Lanka nephropathy in Sri Lanka; and Uddanam endemic nephropathy in India. As the etiology of these nephropathies is not clear, the term chronic kidney disease of unknown etiology has been used for these nephropathies since the early 2000s.^3^Although chronic kidney disease has been recognized as a public health problem, Uddanam endemic nephropathy, compared to the other nephropathies of unknown etiology, is the least understood and the least publicized.^1^

Unpublished cross-sectional estimates from Uddanam suggest that the prevalence of chronic kidney disease of unknown etiology is between 40% and 60% (T Raviraju, Dr, NTR University of health sciences, personal communication, August 2017). This range is nearly three times higher than the national prevalence of 17.2%.^1^^,^^4^ As of 2015, it was estimated that more than 4500 people had died from chronic kidney disease in the last ten years and around 34 000 people had kidney diseases in Uddanam.^1^^,^^5^

Many scientific communities are exploring the causes of chronic kidney disease of unknown etiology in Uddanam region.^6^ Various institutes and organizations such as the Indian Council of Medical Research along with Harvard University, King George Hospital, Andhra University, Dr NTR University of Health Sciences and others are looking into the possible causes of the disease. These institutes have sampled patients’ blood and urine, tested soil, water and food, and surveyed and mapped the population of the affected region. Several hypotheses, such as high levels of silica in water, prolonged dehydration, heat stress nephropathy, nonsteroidal anti-inflammatory drug use, gene mutations, high pesticide use, heavy metals in water and others have been suggested as possible causes. However, until now none of these can explain why the disease is so rampant in Uddanam (T Raviraju, Dr, NTR University of health sciences, personal communication, August 2017).

Lessons learnt from other regional nephropathies could facilitate more focused research. For instance, the Sri Lankan nephropathy study^1^ is notable for its methodological thoroughness. This study involved the use of global positioning devices and various epidemiologic tools, such as stratified random and hot spot sampling. The researchers used sensitive analytic techniques to measure inorganic and organic chemicals including persistent organic pollutants in a variety of biological samples to delineate differences between endemic and non-endemic regions.^1^ The cause was found to be the mixing of brackish waters with sub-standard fertilizers and agrochemicals. In 2014, in collaboration with the World Health Organization (WHO) Sri Lanka Country Office, the Government of Sri Lanka set up a presidential task force to provide oversight and coordinate the efforts of various sectors, agencies and ministries towards the prevention and treatment of chronic kidney diseases.^7^ Prevention is carried out by providing clean drinking water, promoting organic farming of native Sri Lankan rice and banning certain agrochemicals. These measures reduced kidney disease and related deaths.^3^^,^^8^^,^^9^

Uddanam nephropathy is a public health issue in India and sharing expertise across disciplines and countries is needed to accelerate knowledge dissemination, guide the research agenda and help establish its causes. However, it is challenging for India to engage in sustained research, given constrained resource, research capabilities and national policy initiatives. WHO could support these efforts by contributing to reshaping the research agenda and calling for collaboration between clinicians, researchers, epidemiologists, toxicologists, agriculture scientists, social scientists, hydrologists and governmental and nongovernmental organizations, including those who work for the Epidemic Intelligence Service. Local and national governments should strengthen the implementation of available interventions for early detection and management of chronic kidney disease of unknown etiology. They should also prioritize the provision of safe drinking water and food in affected areas and promote sustainable agricultural practices based on current knowledge and evidence until researchers have established the exact cause of this chronic disease. Paramedical personnel trained in renal care, as well as social workers, should also provide social and psychological support to patients, families and communities. All these interventions should be regularly evaluated to assess their impact and adapt them if needed.

If policy-makers do not undertake such steps until a cause is established, people of Uddanam are potentially more at risk of acquiring the disease and patients already having chronic kidney disease of unknown etiology will be at risk of death. The experience of Sri Lanka shows that the scientific community needs to collect data in a comprehensive manner and analyse these thoroughly to increase the chances of finding the cause of Uddanam nephropathy.
